# P-333. Teaching Hand Hygiene Technique to Preschool-aged Students Using a Musical Mnemonic: A Vanguard Study

**DOI:** 10.1093/ofid/ofae631.536

**Published:** 2025-01-29

**Authors:** Nisha Thampi, Aparna Darbha, Anna Zumbansen, Gilles Comeau, Yves Longtin

**Affiliations:** CHEO, Ottawa, Ontario, Canada; University of Ottawa, Ottawa, Ontario, Canada; University of Ottawa, Ottawa, Ontario, Canada; University of Ottawa, Ottawa, Ontario, Canada; Jewish General Hospital, Montreal, Montreal, QC, Canada

## Abstract

**Background:**

The World Health Organization (WHO) recommends a 6-step protocol for hand hygiene (HH). A musical mnemonic, with instructions sung before completion of each step, was developed in 2019 to increase awareness of HH technique. The primary objective of this study was to determine whether the musical mnemonic improves HH technique compared to prose instruction among preschool-aged children through a novel set of process and outcome measures. The secondary objective was to assess feasibility of rolling out the musical mnemonic as part of HH education at a class level.

**Figure:**
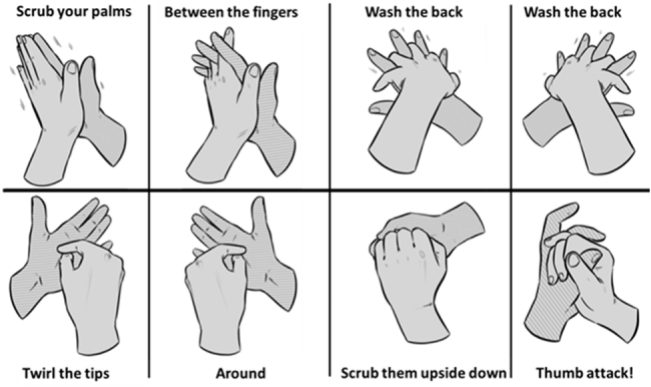

Poster demonstrating hand hygiene technique with instructions according to musical mnemonic

**Methods:**

We partnered with a local school and co-designed a vanguard trial involving students in toddler (age 1-2 years) and preschool (age 3-5 years) classrooms. In all rooms, we introduced the HH technique using conventional teaching, i.e., visual demonstration using a poster with written instructions (Figure 1) and lotion visible under ultraviolet light. Intervention rooms had additional musical mnemonic, or lyrical, instructions. The demonstration was repeated one week later. Four classes (2 toddlers and 2 preschool) were randomized to either conventional or lyrical teaching and followed on Day 1, 2, 5, 8, 9, 22 and 29 to assess the immediate and long-term impact on the degree of completion of the HH steps (scores from 0 to 9).

**Figure:**
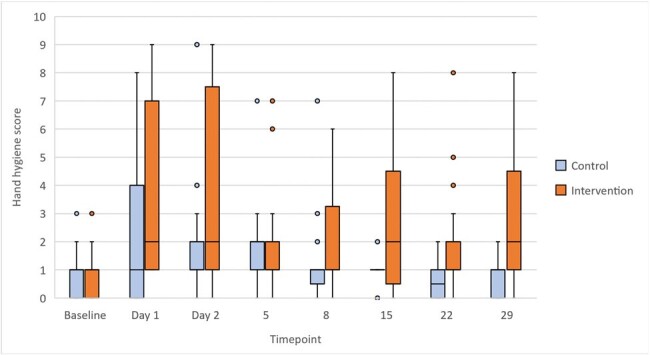

Overall impact of hand hygiene teaching on hand hygiene score (combined toddler and preschool age groups)

**Results:**

We performed 378 HH observations in January and February, 2024. Both lyrical and conventional instruction groups were comparable in terms of HH score at baseline (median [IQR], 1 [0-1] vs 1 [0-1], respectively, *p*=0.90). Immediately after training, the lyrical group achieved a significantly better HH score than the conventional group (median [IQR], 2 [1-7] vs 1 [0-4], *p*=0.007). The effect of the intervention was sustained, with significantly higher HH scores in both toddler and preschool age groups on Day 2, 15, 22 and 29 after intervention (*p*< 0.05 for each comparison, Figure 2).

**Conclusion:**

A musical mnemonic was associated with an immediate, improved uptake of the WHO-recommended HH technique, then decreased but remained significantly higher compared to conventional teaching over 4 weeks among preschool- and toddler-aged students. A multimodal HH education program that incorporates the musical mnemonic can be co-designed with education specialists and evaluated in a school setting.

**Disclosures:**

**All Authors**: No reported disclosures

